# Effects of an mHealth Intervention for Pulmonary Tuberculosis Self-management Based on the Integrated Theory of Health Behavior Change: Randomized Controlled Trial

**DOI:** 10.2196/34277

**Published:** 2022-07-14

**Authors:** Yuhan Bao, Chunxiang Wang, Haiping Xu, Yongjie Lai, Yupei Yan, Yuanyuan Ma, Ting Yu, Yibo Wu

**Affiliations:** 1 Health Clinic, Changzhou Institute of Technology Changzhou China; 2 School of Sciences, Changzhou Institute of Technology Changzhou China; 3 School of Pharmaceutical Sciences, Shandong University Jinan China; 4 Department of Humanities, Arts and Media, Changzhi Medical College Changzhi China; 5 School of Public Health, Shandong University Jinan China; 6 School of Public Health, Peking University Beijing China; 7 Key Research Base of Philosophy and Social Sciences in Shaanxi Province, Health Culture Research Center of Shaanxi Xi'an China

**Keywords:** ITHBC, mHealth, RCT, pulmonary tuberculosis

## Abstract

**Background:**

Improving the health self-management level of patients with tuberculosis (TB) is significant for reducing drug resistance, improving the cure rate, and controlling the prevalence of TB. Mobile health (mHealth) interventions based on behavioral science theories may be promising to achieve this goal.

**Objective:**

This study aims to explore and conduct an mHealth intervention based on the Integrated Theory of Health Behavior Change (ITHBC) in patients with pulmonary TB to increase their ability of self-care management.

**Methods:**

A prospective randomized controlled study was conducted from May to November 2020. A total of 114 patients who were admitted consecutively to the TB clinic of Harbin Chest Hospital, China from May 2020 to August 2020 were recruited by convenience sampling. Patients were divided into the control group and intervention group, and all received a 3-month intervention. Patients in the intervention group and the control group received routine medical and nursing care in the TB clinic, including the supervision of their medications. In addition, pharmacist-assisted mHealth (WeChat) intervention based on the ITHBC theory about TB management was provided to the intervention group. The primary outcome was self-management behavior, while the secondary outcomes were TB awareness, self-efficacy, social support, and degree of satisfaction with health education. The outcomes were measured using web-based self-designed and standard questionnaires administered at baseline and at the end point of the study. Intergroup data were assessed using the Mann-Whitney *U* test, whereas intragroup data were assessed with the Wilcoxon test (for paired samples).

**Results:**

A total of 112 patients (59 in intervention group and 53 in control group) completed the study. After the intervention, a statistically significant increase was noted in the scores of each item of self-care management behaviors compared with the scores at the baseline (*P*<.001) in the intervention group. The scores of all self-care management behaviors of the control group were lower than those of all self-care management behaviors in the intervention group (all *P*<.05), except for the item “cover your mouth and nose when coughing or sneezing” (*P*=.23) and item “wash hands properly” (*P*=.60), which had no statistically significant difference from those in the intervention group. Compared with those at baseline, TB knowledge awareness, self-efficacy, social support, and degree of satisfaction with health education in the intervention group increased significantly (*P*<.001), and the intervention group had significantly higher scores than the control group (*P*<.001).

**Conclusions:**

mHealth intervention for TB self-management based on ITHBC could deepen the understanding of patients with TB about their disease and improve their objective initiative and self-care management behaviors, which were beneficial for promoting compliance behavior and quality of prevention and control for pulmonary TB.

**Trial Registration:**

Chinese Clinical Trial Registry ChiCTR2200055557; https://tinyurl.com/4ray3xnw

## Introduction

Tuberculosis (TB) is a communicable disease, which is one of the top 10 causes of death worldwide and the leading cause of death from a single infectious agent (ranking above AIDS). According to the estimates of the World Health Organization, in 2019, there were 9.87 million new cases of TB and 1.28 million deaths. China is one of the 30 high TB burden countries. In 2020, the number of new TB cases in China was 842,000, ranking second in the world [1]. Hence, TB is a major public health problem with high incidence and mortality worldwide.

Self-management is defined as a task that patients undertake to deal with the medical, role, and emotional management of their chronic conditions [2]. For patients with TB, self-management includes adhering to medication and treatment, maintaining a healthy diet and adequate amount of exercise, keeping a good mental state, and strengthening personal capacity to solve problems [3]. Therefore, improving the self-management level of patients with TB is of great significance for controlling their illness, increasing their quality of life, improving the cure rate, and controlling the prevalence of TB [4].

Public health programs have used various interventions to improve the self-management level of patients with TB, especially with regard to their adherence to TB treatment [5]. One of the most common interventions is directly observed therapy (DOT), in which a health worker, family member, or community member observes the patient taking TB medications [6]. The fact that people can be closely monitored and the social process with peer pressure are the advantages of DOT that may improve patients’ medication adherence [7]. However, until now, DOT is still not optimal because of its inconvenience and labor-intensive practice [8,9]. Moreover, the COVID-19 pandemic has resulted in the need for “social distancing,” which has caused the suspension of DOT and an exponential use of mobile health (mHealth) approaches for patient care [10]. Further, the World Health Organization has called for maximizing remote care and support for people with TB by expanding the use of digital technologies [1]. All these factors have driven the development of mHealth that can be defined as using mobile computing and communication technologies in support of health care to supplement the traditional clinical-based care [11]. Common mHealth interventions and programs include video-based DOT and mobile phone text messaging to support treatment compliance and health education [9]. So far, the acceptability, feasibility, and efficiency of mHealth in improving patient adherence and supporting TB treatment have shown promising results [12]. This study will take advantage of mHealth and make use of web-based health interventions for patients.

**Figure 1:**
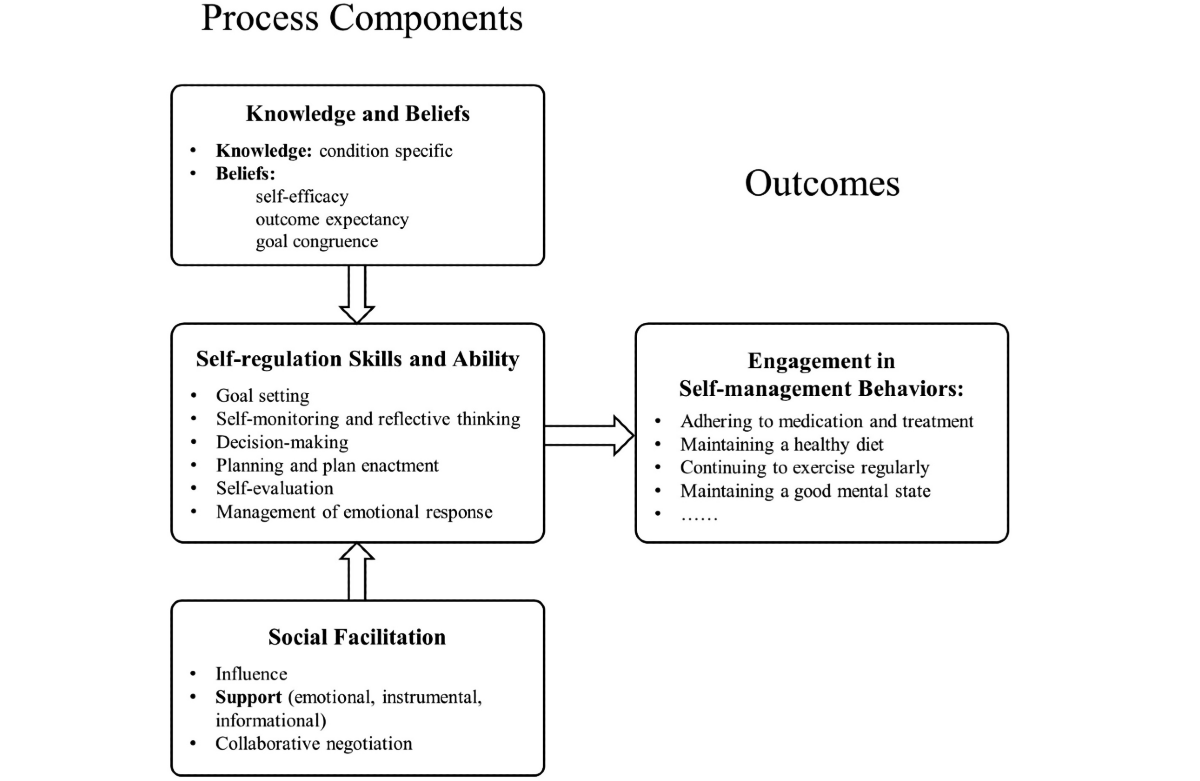
The theoretical framework of Integrated Theory of Health Behavior Change.

Although evidence indicates that health promotion interventions based on behavioral science theories are more effective than those without theoretical models, only few interventional studies related to TB self-management have used theoretical models as guidance [13]. Therefore, we aimed to evaluate an mHealth intervention to improve the self-management level of patients with TB based on the Integrated Theory of Health Behavior Change (ITHBC). The ITHBC suggests that health behavior change can be promoted by enhancing knowledge and beliefs, strengthening social facilitation, and increasing self-regulation skills and abilities, of which the first two promote the latter [14]. Engagement in self-management behaviors is regarded as the proximal outcome influencing the distal outcome of improved health status [15]. The ITHBC summarizes the key components of health behavior change processes and provides the pivotal components for intervention development, which has great referential significance for the design of intervention measures in this study. The theoretical framework of ITHBC is shown in Figure 1.

WeChat is the most popular social media platform in China [16]. Its wide application in the daily life of every Chinese indicates that it a promising new medium for delivering health care in a cost-effective way. Accumulating evidence has robustly proved that WeChat-based mHealth is acceptable, feasible, and cost-effective in improving health outcomes in various health conditions [17,18]. Therefore, we aimed to develop an ITHBC-based mHealth intervention targeting self-management in patients with TB and to study whether this WeChat-based intervention can significantly improve the self-management level of patients with TB and the cure rate of TB and reduce the infection rate and drug resistance.

## Methods

### Study Design

A prospective randomized controlled trial was conducted in Harbin Chest Hospital, China from May to November 2020. Convenience sampling was performed to recruit 114 patients with pulmonary TB. They were assigned into the intervention group and control group in a 1:1 ratio by using a computer-based random number generator, and all received a 3-month intervention. WeChat groups were created by a pharmacist to provide health education for the participants. Patients in the intervention group and the control group received routine medical and nursing care in the TB clinic, including the supervision of their medications. In addition, pharmacist-assisted mHealth intervention based on the ITHBC theory about TB management was provided to the intervention group. This trial is reported in accordance with CONSORT-eHEALTH (see Multimedia Appendix 1 for the completed CONSORT-eHEALTH form V 1.6.1).

Based on the ITHBC theory, we summarized the epidemical characteristics of TB and the self-management and psychology of patients with TB through a literature review. The research team created a multidisciplinary panel comprising of a TB pharmacist, 2 nursing experts, 2 TB physicians, and a public health expert to discuss and develop the intervention program. A statistician was also included to be responsible for the random allocation of the participants and data analysis. Semistructured interviews were conducted with 4 patients randomly selected in the finally included participants to know their mastery of TB knowledge, diets, exercise habits, risk factors and awareness, personal needs and wishes, and concerns and inertia. After communication, we conducted individualized assessments to refine the intervention program. Finally, 20 participants who would not be enrolled in the final trials were randomly selected to complete the pretest to refine self-designed scales. The face and content validity of the self-designed questionnaire were determined by pretest and experts, respectively.

### Sample Size

The formula for sample size calculation was as follows:



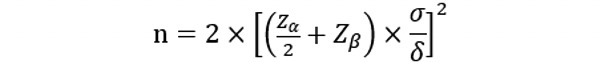



We assumed *σ*=1.6 and *δ*=1.02 with reference to a preliminary study [19] when *α*=1.6, 1-*β*=0.80, and then we got n=40 for 1 group. Considering dropouts (20%) over the course of the study, 100 patients were included (50 for control group and 50 for intervention group).

### Study Participants

The participants in this study were patients who were admitted consecutively to the TB clinic of Harbin Chest Hospital from May to August 2020, and the study was conducted between May and November 2020. Each participant’s intervention started as long as they were enrolled offline at the hospital by face-to-face communication. Therefore, all the patients were not recruited or started with the intervention at the same time, but all completed the 3-month intervention.

The eligibility criteria were (1) 18 years old or older, (2) experiencing newly diagnosed active pulmonary TB according to the classification of TB (WS 196-2017) issued by the People’s Republic of China State Health and Family Planning Commission [20], (3) literate and capable of using WeChat, and (4) hospitalized when enrolled in the study. The exclusion criteria were as follows: (1) experiencing drug-resistant pulmonary TB or treated according to the treatment plan of drug-resistant TB and (2) complicated with serious diseases such as AIDS, malignant tumor, and severe diseases that were newly diagnosed in the heart, brain, liver, and kidney. All patients in both the groups received the therapy in the hospital for nearly 1 month since the intervention started and then received TB outpatient treatment for 2 months. The median hospitalization time for the intervention group was 25 (IQR 18-32) days, while that for the control group was 24 (IQR 16-31) days (*P*=.35). All patients gave web-based informed consent to participate in this study (see Multimedia Appendix 2 for the informed consent). Patients whose treatment regimens were changed or lost to follow-up were terminated from this study.

### Interventions

**Figure 2:**
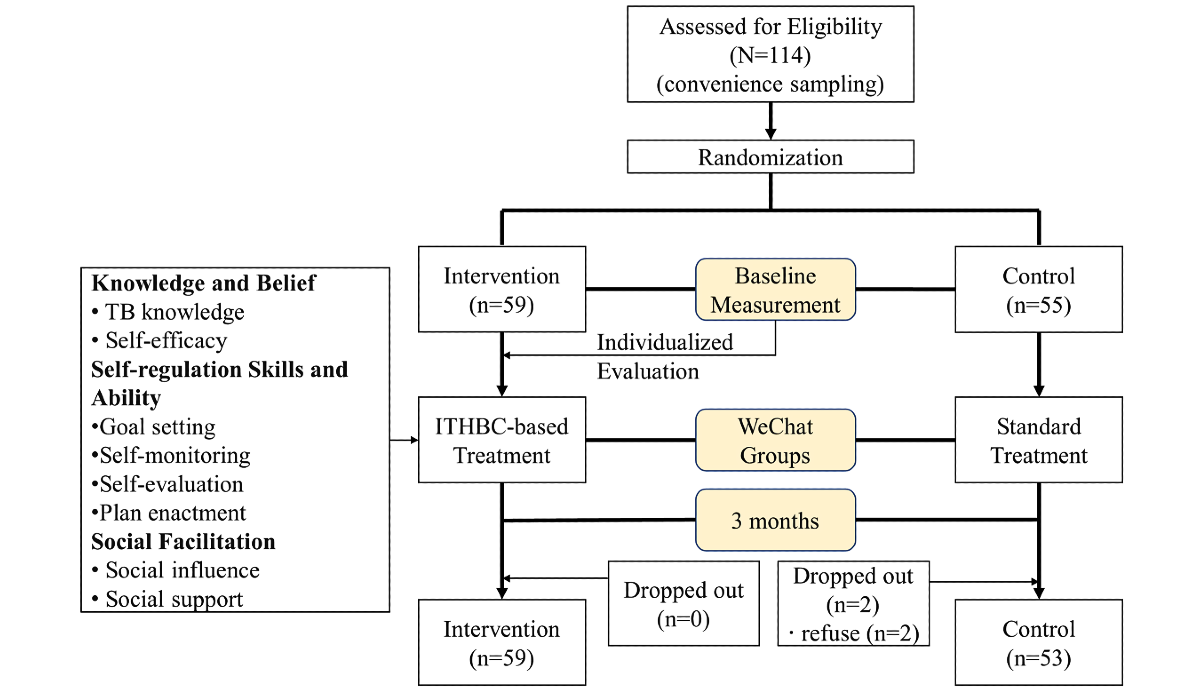
The flow chart of the randomized controlled trial. ITHBC: Integrated Theory of Health Behavior Change; TB: tuberculosis.

Intervention for the intervention group on the day of hospitalization included health education in WeChat groups, web-based health education lectures, and receiving health education plans as well as routine medical and nursing care such as supervision of their medications, which continued for 1 month. Then they received therapy at outpatient clinics and were provided with the same intervention as provided in the hospital for 2 months (see Multimedia Appendix 3 for the screenshots of the health education in the WeChat group). Patients in the control group received routine medical and nursing care in the TB clinic, including supervision of their medications. Meanwhile, the pharmacist also created a WeChat group for the control group to allow patients to communicate with each other but without any intervention. The content of the intervention was designed based on the ITHBC theory, and the flow chart is presented in Figure 2.

### Knowledge and Belief

Information and desire to change are the prerequisites that patients engage in recommended behaviors. In our study, health education can be divided into knowledge education and belief education. Knowledge was defined as information of pulmonary TB, and belief was defined as personal perceptions about patients’ health condition or health behaviors [14]. Knowledge education contained basic knowledge, diet management, hygiene routines, and medication management. Belief education was conducted in weekly meetings to increase the consciousness of crisis for disease and patient confidence for disease management. The pharmacist gave individualized health education based on baseline information. Education forms included WeChat groups and online lectures. The topics of the health education are presented in Multimedia Appendix 4, which were sent to the WeChat group in the form of articles, pictures, or videos. Patients were guided every Monday for 10 minutes in WeChat. Web-based lectures were conducted every Thursday for an hour through WeChat.

### Self-regulation Skills and Ability

Patients with the desire to change health behaviors should learn self-regulation skills and the ability to apply TB knowledge to their lives. To improve self-regulation skills and ability, the pharmacist summarized patients’ current behavior and issues measured at the baseline to make an individualized health education plan in a month and a short-term aim in a week during hospitalization. The plans in the latter 2 months would be made according to performance of the patients in the previous month. Patients were asked to self-monitor and record daily health behaviors and emotions. A meeting was held by the pharmacist in the WeChat group every Saturday, where patients reported and self-evaluated their own behaviors and emotions; moreover, they had exchange of views with wardmates. The pharmacist would give some timely guidance to help the patients make the next aim themselves.

### Social Facilitation

Excellent social facilitation positively influences and supports patients’ engagement in health behaviors. According to the ITHBC theory, social facilitation includes social influence and social support. Patients are more likely to engage in health behaviors when authorities sway their thinking and motivation, which is called social influence. In our study, social influence came from health care providers, especially pharmacists who offered the intervention, family, and significant others (eg, wardmates, friends, neighbors, colleagues, social media). Social support includes emotional, instrumental, and informational support, which were offered by health care providers, family, friends, and others. The patient’s family received health education in rounds with patients, attended web-based lectures, and supervised the patients. They were encouraged to take the lead in adopting health behaviors and provide comfort and company for the patients in their family. The pharmacist provided instrumental and informational support by establishing a WeChat group and health education. Wardmates communicated with and encouraged each other in wards and in the WeChat group. During the intervention, when the country was in lockdown during the COVID-19 epidemic, patients also received much tutorial advice on preventive behaviors, as the coronavirus transmission is similar to that of pulmonary TB virus.

### Outcome Measures

All the patients filled out a web-based questionnaire of demographic information at baseline (see Multimedia Appendix 5 for the questionnaire 1). The primary outcome was self-management behavior. Secondary outcomes were TB knowledge awareness, self-efficacy, social support, and degree of satisfaction with health education. The outcome data were collected by scales at baseline and at the end of the study when patients were hospitalized and at home, respectively. Clinical pharmacists used the web-based platform of Wen Juan Xing [21] to distribute the questionnaire (see Multimedia Appendix 5 for the questionnaire 2), and patients filled it while the pharmacist guided them face-to-face (in hospitalization) or telephone (after discharge). The demographic information of the patients was collected by the medical record system and the self-designed questionnaire.

### Primary Outcome: Self-management Behavior

Self-management behavior was measured using a self-designed structured scale with item 4 in questionnaire 2. General self-management behaviors consisted of COVID-19-related behaviors, medication behaviors, and lifestyle habits with 13 items and a 5-level Likert scoring method. Each item was scored as 1-4 points, with 1=no intention to act, 2=intention to act, 3=having acted but stopped, 4=starting to act, and 5=having acted and considering keeping on. Higher scores indicated greater self-management behaviors. Cronbach *α* was .966, suggesting good internal consistency of the scale.

### Secondary Outcomes

#### TB Knowledge Awareness

A self-designed constructed questionnaire (item 3) was administered to evaluate TB knowledge awareness in basic knowledge and hygiene routines with 5 items and a 5-level Likert scoring method. Answers were comprised of totally disagree (score=1), disagree (score=2), uncertain (score=3), agree (score=4), and totally agree (score=5). The score of each item was summed to give a total TB knowledge score, with higher score indicating a better knowledge level. The coefficient of Cronbach *α* was .772.

#### Self-efficacy

The chronic disease self-efficacy scale [22] (items 6-11) was applied to measure the self-efficacy of patients. This scale has 6 items, including symptom management, daily life management, emotional management, and disease control. A 10-point scoring test was used, and each item was rated from 1=not at all confident to 10=completely confident. The score of each item was summed to give the scale’s score (range 6-60), and higher scores indicated higher self-efficacy level. The coefficient of Cronbach *α* was .910.

#### Social Support

The perceived social support scale, designed by Blumenthal et al [23] (item 2), was used to assess a patient’s perception of the social support from family, friends, and significant others. The scale had 12 items and contained 3 subscales of 4 items each. Response options ranged from 1=totally dissatisfied to 7=totally satisfied. Higher scores indicated more social support. The coefficient of Cronbach *α* was .97.

#### Degree of Satisfaction With Health Education

To assess patients’ satisfaction of the intervention organized by the pharmacists, a self-designed scale (item 5) based on the Unified Theory of Acceptance and Use of Technology [24] was applied to evaluate the degree of satisfaction with health education. This item had 10 subitems and consisted of 3 sections, which are performance expectancy (4 subitems), effort expectancy (4 subitems), and facilitating conditions (2 subitems). It was a 7-point scale from “totally dissatisfied” to “totally satisfied.” A higher score indicated higher satisfaction. The coefficient of Cronbach *α* was .963.

### Quality Control

In order to ensure the quantity and homogeneity of the participants, the participants were selected in strict accordance with the criteria of inclusion and exclusion, which were checked by TB physicians. We chose newly diagnosed patients with active pulmonary TB to make sure of a consistent treatment plan and to reduce bias caused by different medication therapies. A study [25] has shown that the generation of a random sequence should be done by some independent personnel, usually a statistician, who is not going to be involved in the conduct of the randomized controlled trial. To ensure the scientific nature of the random allocation, this study had a statistician responsible for the random allocation of the participants and the data analysis. The researchers discussed the intervention measures of this study with an interdisciplinary panel in the field of TB and conducted structured interviews with the participants in order to further improve the intervention program. In addition, a pretest was conducted before formal intervention to find the problems in the design of the intervention and to optimize the intervention program. The clinical pharmacist explained the purpose and significance of this study to the participants and obtained informed consent. In the process of filling out the questionnaire, the clinical pharmacist helped to explain the questionnaire to the patients to ensure that the questionnaire was completed efficiently, but it was not instructive. Moreover, when using the data collection tool, known as Wen Juan Xing, patients could not submit the questionnaire unless they completed every question, which avoided missing data.

### Statistical Analysis

Statistical analysis was implemented using SPSS Statistics 26.0 software (IBM Corp). Normality tests were applied to assess the distribution of the continuous data, and nonnormally distributed data were presented as median (IQR). The Mann-Whitney *U* test was used to compare the changes in each scale score between the 2 groups at the baseline and the end point, while the Wilcoxon test (for paired samples) was applied to compare the difference between the baseline and the end point of each scale score in each group. The demographic characteristics of the 2 groups were compared using the chi-square test. *P*<.05 (2-tailed test) was considered as statistically significant.

### Ethics Approval

This study was approved by the ethics committee of Harbin Chest Hospital, China (2020-10).

## Results

### Characteristics of the Patients

A total of 114 patients participated in this study, and they were randomly assigned to the intervention group (n=59) and the control group (n=55). During the study period, 2 patients dropped out (2/114, 1.8%). Therefore, 112 patients were included in the statistical analysis finally. Of the 112 patients, 64 (57.1%) were males. All patients were aged 18 years and older and were mainly aged between 18 and 30 years (39/112, 34.8%), and only 7 (6.3%) were older than 60 years. Approximately half of the patients had a college degree or higher (51/112, 45.5%). Most patients (101/112, 90.2%) lived with their families or friends, and 71 (63.4%) were married. The demographic information of the patients is shown in Table 1. At baseline, approximately all patients’ characteristics were similarly distributed between the intervention and the control groups.

**Table 1:** Demographic characteristics of the patients.

Characteristics		Intervention group (n=59), n (%)	Control group (n=53), n (%)	*χ^2^ (df)*		*P* value	
**Gender**					1.1 (1)		.30
	Female	31 (53)	33 (62)				
	Male	28 (47)	20 (38)				
**Age group (years)**					1.7 (4)		.79
	18-30	21 (36)	18 (34)				
	31-40	15 (25)	12 (23)				
	41-50	7 (12)	11 (21)				
	51-60	12 (20)	9 (17)				
	>60	4 (7)	3 (6)				
**Race/ethnicity**					0 (1)		.93
	Han	58 (98)	51 (96)				
	Other ethnicities	1 (2)	2 (4)				
**Religious belief**					0 (1)		>.99
	There is belief	4 (7)	4 (8)				
	No belief	55 (93)	49 (92)				
**Marital status**					1.9 (2)		.40
	Unmarried	18 (31)	19 (36)				
	Married	40 (68)	31 (58)				
	Others	1 (2)	3 (6)				
**Per capita monthly income (CNY)^a^**					2.8 (5)		.74
	<1000	8 (14)	11 (21)				
	1000-1999	8 (14)	9 (17)				
	2000-2999	16 (27)	13 (25)				
	3000-3999	15 (25)	13 (25)				
	4000-4999	4 (7)	1 (2)				
	≥5000	8 (14)	6 (11)				
**Usual residence**					1.4 (1)		.23
	Rural	12 (20)	16 (30)				
	Urban	47 (80)	37 (70)				
**Education**					0.7 (3)		.88
	Primary school and below	3 (5)	3 (6)				
	Junior middle school	14 (24)	10 (19)				
	High school/technical secondary school	17 (29)	14 (26)				
	College and above	25 (42)	26 (49)				
**Residence status**					0.6 (1)		.44
	Alone	7 (12)	4 (8)				
	Others	52 (88)	49 (92)				
**Relatives or friends were infected by tuberculosis**					0 (1)		>.99
	Yes	5 (8)	5 (9)				
	No	54 (92)	48 (91)				

^a^1 CNY=US $0.14.

### Primary Outcome: Self-management Behavior

**Figure 3:**
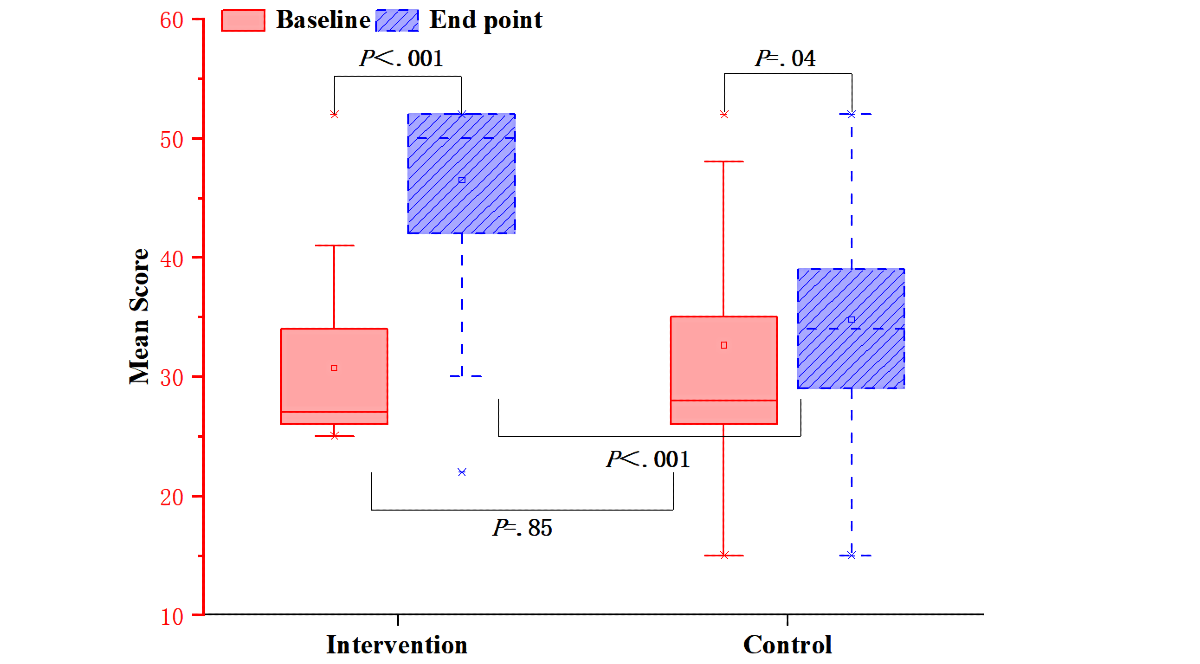
Mean scores of self-management behaviors for both groups before and after the intervention.

The total scores of all the 13 self-management behaviors were calculated. Both groups scored greater than that at baseline (*P*<.001 vs *P*=.04, respectively), but the total score of the intervention group was higher than that of the control group (*P*<.001) (Figure 3). Table 2 shows that there was no significant difference in the scores of all self-management behaviors between the intervention group and the control group at baseline.

After the intervention, there were significant differences in the scores of the above behaviors between the 2 groups except for “covering your mouth and nose when coughing or sneezing” (*P*=.23) and “wash hands properly” (*P*=.60) (Table 3).

The scores of self-management of health behaviors in the intervention group increased significantly after the intervention (*P*<.001). The 4 health behaviors with significant increases in the scores in the control group were “stay in a good mood” (*P*=.002), “maintain a balanced diet and ensure the intake of high protein, vitamins, and minerals” (*P*=.002), “cover your nose and mouth when coughing or sneezing” (*P*<.001), and “wash hands properly” (*P*<.001). Although the score of the item “self-isolate from families in home-based treatment” was lower than that at the baseline (*P=*.01), there was no significant change in the scores of other behaviors in the control group (Table 4).

**Table 2:** Scores of self-management health behaviors at baseline in the intervention and control groups.

Self-management behaviors	Intervention group			Control group			*Z*		*P* value
	Median (P25-P75)	Mean	Median (P25-P75)		Mean				
(1) Self-isolate from families in home-based treatment	2.00 (1.00-2.00)	1.88	2.00 (1.00-2.00)		1.91	–0.593		.55	
(2) Keep your room well-ventilated	2.00 (2.00-2.00)	2.25	2.00 (2.00-3.00)		2.49	–1.513		.13	
(3) Maintain a balanced diet and ensure enough intake of high protein, vitamins, and minerals	2.00 (2.00-3.00)	2.36	2.00 (2.00-3.00)		2.47	–0.575		.57	
(4) Stick to daily exercise	2.00 (2.00-2.00)	2.24	2.00 (2.00-3.00)		2.47	–1.519		.13	
(5) Go to bed and wake up early, and alternate work with rest	2.00 (2.00-2.00)	2.24	2.00 (2.00-2.00)		2.36	–0.565		.57	
(6) Stay in a good mood	2.00 (2.00-3.00)	2.32	2.00 (2.00-3.00)		2.51	–0.879		.38	
(7) Cover your nose and mouth when coughing or sneezing	2.00 (2.00-3.00)	2.44	3.00 (2.00-3.00)		2.70	–1.794		.07	
(8) Choose and wear a mask rightly	2.00 (2.00-3.00)	2.54	3.00 (2.00-4.00)		2.87	–1.723		.09	
(9) Wash your hands with soap and water for at least 20 seconds regularly	2.00 (2.00-3.00)	2.61	3.00 (2.00-4.00)		2.87	–1.379		.17	
(10) Adopt disinfection measures (eg, 70% alcoholic solutions or 1% sodium hypochlorite solution) in daily life	2.00 (2.00-3.00)	2.47	2.00 (2.00-3.00)		2.42	–0.799		.42	
(11) Focus on your symptoms and adverse drug reactions	2.00 (2.00-3.00)	2.39	2.00 (2.00-3.00)		3.45	–0.08		.94	
(12) Do not change or stop medication easily	2.00 (2.00-3.00)	2.46	2.00 (2.00-3.00)		2.55	–0.422		.67	
(13) Reexamine regularly	2.00 (2.00-3.00)	2.51	2.00 (2.00-3.00)		2.55	–0.027		.98	

**Table 3:** Scores of self-management health behaviors at the end point of the study in the intervention and control groups.

Self-management behaviors	Intervention group		Control group		*Z*	*P* value
	Median (P25-P75)	Mean	Median (P25-P75)	Mean		
(1) Self-isolate from families in home-based treatment	4.00 (2.00-4.00)	3.03	1.00 (1.00-2.00)	1.60	–5.308	<.001
(2) Keep your room well-ventilated	4.00 (4.00-4.00)	3.44	2.00 (1.00-4.00)	2.53	–3.843	<.001
(3) Maintain a balanced diet and ensure enough intake of high protein, vitamins, and minerals	4.00 (3.00-4.00)	3.47	4.00 (2.00-4.00)	3.04	–2.055	.04
(4) Stick to daily exercise	4.00 (2.00-4.00)	3.14	2.00 (1.00-4.00)	2.30	–3.096	.002
(5) Go to bed and wake up early, and alternate work with rest	4.00 (4.00-4.00)	3.53	2.00 (1.50-4.00)	2.51	–4.601	<.001
(6) Stay in a good mood	4.00 (3.00-4.00)	3.51	3.00 (2.00-4.00)	3.02	–3.246	.001
(7) Cover your nose and mouth when coughing or sneezing	4.00 (4.00-4.00)	3.75	4.00 (4.00-4.00)	3.60	–1.213	.23
(8) Choose and wear a mask rightly	4.00 (4.00-4.00)	3.80	4.00 (1.50-4.00)	3.17	–2.809	.005
(9) Wash your hands with soap and water for at least 20 seconds regularly	4.00 (4.00-4.00)	3.78	4.00 (4.00-4.00)	3.70	–0.522	.60
(10) Adopt disinfection measures (eg, 70% alcoholic solutions or 1% sodium hypochlorite solution) in daily life	4.00 (4.00-4.00)	3.44	1.00 (1.00-3.50)	2.02	–5.595	<.001
(11) Focus on your symptoms and adverse drug reactions	4.00 (4.00-4.00)	3.75	2.00 (1.00-4.00)	2.21	–6.416	<.001
(12) Do not change or stop medication easily	4.00 (4.00-4.00)	3.73	3.00 (2.00-4.00)	2.83	–4.566	<.001
(13) Reexamine regularly	4.00 (4.00-4.00)	3.61	2.00 (1.00-4.00)	2.23	–5.783	<.001

**Table 4:** Intragroup comparison results for the self-management health behaviors (end point–baseline).

Self-management behaviors	Intervention group			Control group		
	Mean difference	*Z*	*P* value	Mean difference	*Z*	*P* value
(1) Self-isolate from families in home-based treatment	1.15	–4.828	<.001	–0.30	–2.544	.01
(2) Keep your room well-ventilated	1.19	–5.376	<.001	0.04	–0.576	.57
(3) Maintain a balanced diet and ensure enough intake of high protein, vitamins, and minerals	1.12	–5.038	<.001	0.57	–3.166	.002
(4) Stick to daily exercise	0.90	–4.568	<.001	–0.17	–0.582	.56
(5) Go to bed and wake up early, and alternate work with rest	1.29	–5.639	<.001	0.15	–1.251	.21
(6) Stay in a good mood	1.19	–5.602	<.001	0.51	–3.050	.002
(7) Cover your nose and mouth when coughing or sneezing	1.31	–5.442	<.001	0.91	–4.647	<.001
(8) Choose and wear a mask rightly	1.25	–5.323	<.001	0.3	–1.413	.16
(9) Wash your hands with soap and water for at least 20 seconds regularly	1.17	–5.324	<.001	0.83	–4.358	<.001
(10) Adopt disinfection measures (eg, 70% alcoholic solutions or 1% sodium hypochlorite solution) in daily life	0.97	–4.863	<.001	–0.40	–1.889	.06
(11) Focus on your symptoms and adverse drug reactions	1.36	–5.917	<.001	–0.25	–1.157	.25
(12) Do not change or stop medication easily	1.27	–5.648	<.001	0.28	–1.949	.051
(13) Reexamine regularly	1.10	–5.328	<.001	–0.32	–1.764	.08

### Secondary Outcomes

#### TB Knowledge Awareness

**Figure 4:**
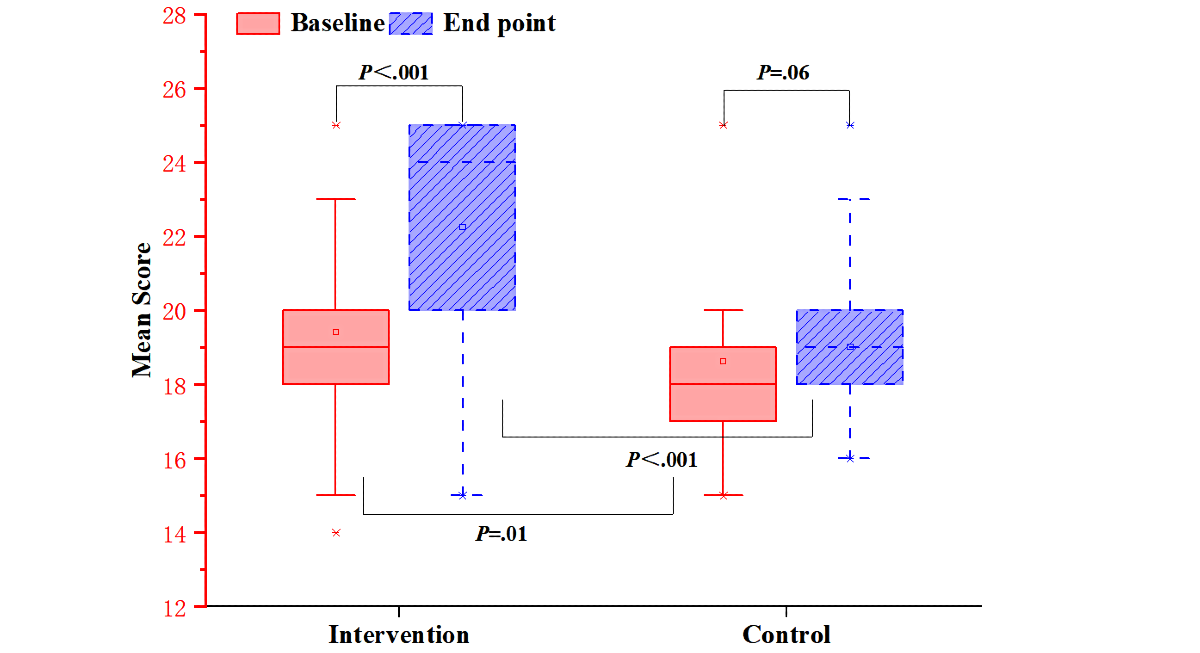
Mean scores of tuberculosis knowledge awareness for both groups before and after the intervention.

The score of TB knowledge awareness in the intervention group increased significantly after the intervention (*P*<.001), but there was no significant differences in the control group between that at baseline and that at the end point (*P*=.06) (Figure 4).

#### Self-efficacy

**Figure 5:**
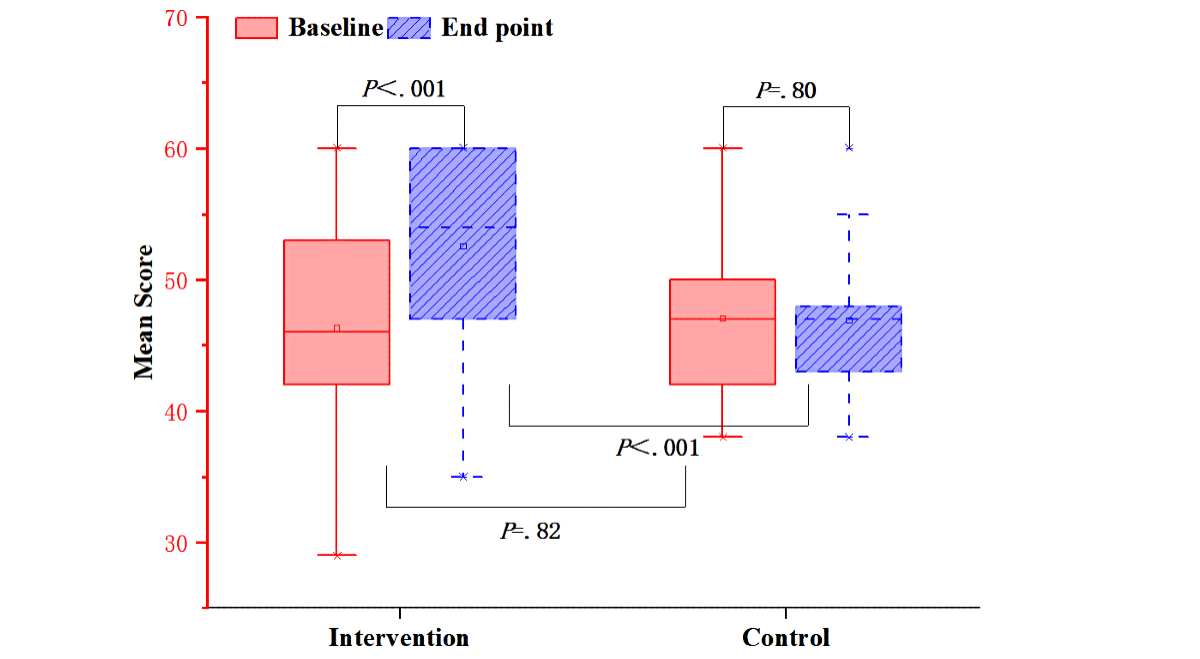
Mean scores of self-efficacy for both groups before and after the intervention.

After the intervention, the score of self-efficacy was significantly improved in the intervention group (*P*<.001), but there was no significant change in the control group between that at baseline and that at the end point (*P*=.80). The score of the intervention group was significantly higher than that of the control group (*P*<.001) (Figure 5).

#### Social Support

The social support scale includes 3 dimensions: family support, friend support, and other support (from significant others). Although the score of friend support in the control group was significantly higher than that in the intervention group at baseline (*P*=.008), after the intervention, the scores of the 3 dimensions were significantly improved in the intervention group (*P*<.001) and were significantly higher than those in the control group (*P*<.001). There were no significant changes in the scores of the control group before and after the intervention (Table 5 and Table 6).

**Table 5:** Median scores of social support scale for both groups at baseline and end point.

Social support scale		Intervention group (n=59), median (P25-P75)	Control group (n=53), median (P25-P75)	Mean difference	*Z*	*P* value
**Family support**						
	Baseline	24 (22-24)	24 (23-24)	–0.4	–0.983	.33
	End point	24 (23-27)	24 (23-24)	2.566	–5.111	<.001
**Friend support**						
	Baseline	21 (18-24)	24 (20-24)	–1.798	–2.664	.008
	End point	24 (21-25)	24 (20-24)	0.432	–0.817	.41
**Other support**						
	Baseline	23 (21-24)	24 (23-24)	–0.11	–0.05	.96
	End point	24 (22-28)	24 (23-24)	1.444	–3.569	<.001

**Table 6:** Intragroup comparison results for social support scale (end point–baseline).

Group	Family support				Friend support			Other support		
	Mean difference	*Z*	*P* value	Mean difference		*Z*	*P* value	Mean difference	*Z*	*P* value
Intervention (n=59)	2.136	–2.407	.02	2.475		–3.833	<.001	2.61	–3.589	<.001
Control (n=53)	–0.83	–0.255	.80	0.245		–0.809	.42	1.057	–0.115	.91

#### Degree of Satisfaction With Health Education

The scale for degree of satisfaction with health education evaluates performance expectancy, effort expectancy, and facilitating conditions. After the intervention, the scores of the 3 dimensions were significantly improved in the intervention group (*P*<.001) and were significantly higher than those in the control group (*P*<.001). There were no significant changes in the scores of the control group before and after the intervention (Table 7 and Table 8).

**Table 7:** Median scores of the degree of satisfaction with health education for both groups at baseline and end point.

Satisfaction scale		Intervention group (n=59), median (P25-P75)	Control group (n=53), median (P25-P75)	Mean difference	*Z*	*P* value
**Performance expectancy**						
	Baseline	19 (16-22)	20 (19-22)	–0.492	–1.215	.22
	End point	24 (22-28)	19 (17-24)	4.35	–5.22	<.001
**Effort expectancy**						
	Baseline	19 (16-23)	20 (19-24)	–0.781	–1.54	.12
	End point	24 (23-28)	20 (19-24)	3.848	–5.22	<.001
**Facilitating conditions**						
	Baseline	8 (8-12)	10 (8-12)	–0.516	–1.17	.24
	End point	12 (12-14)	10 (7-12)	2.781	–5.607	<.001

**Table 8:** Intragroup comparison results for the degree of satisfaction with health education (end point–baseline).

Group	Performance expectancy			Effort expectancy			Facilitating conditions		
	Mean difference	*Z*	*P* value	Mean difference	*Z*	*P* value	Mean difference	*Z*	*P* value
Intervention (n=59)	4.559	–5.403	<.001	4.441	–5.406	<.001	2.881	–5.591	<.001
Control (n=53)	–0.283	–0.427	.67	–0.189	–0.182	.86	–0.415	–0.988	.32

## Discussion

### Principal Findings

#### Overview

Self-management is crucial for the control of chronic diseases. ITHBC was formed on the basis of integration and absorption of many related health behavior change theory models [14], which were used for guiding the establishment and maintenance of health behaviors, thereby changing poor individual lifestyles to better health status and quality of life. In China, mobile services, especially short message service is ubiquitous, as it can deliver frequent prompts and health information to improve treatment adherence [26-29]. With the emergence of the COVID-19 pandemic and the adoption of public health measures for its containment, forms of health education were affected around the world. mHealth interventions did not have any restrictions during the COVID-19 pandemic and met the needs of the patients to the greatest extent. Moreover, the internet brings strong interactions between health care providers and patients at lower cost. We developed an mHealth intervention program based on the ITHBC theory and evaluated its effects on improving patients’ self-management ability with newly diagnosed pulmonary TB. Such an intervention empowers patients to change poor health behaviors based on disease knowledge, self-efficacy, and social support [30,31].

#### Primary Outcomes

After calculation of the total score of 13 self-management behaviors, we found that the scores of both the groups increased, but the scores of the intervention group increased more than those of the control group. However, the total score could not easily explain the effect of our intervention; therefore, further analysis for each self-management behavior needs to be conducted. After the intervention, a statistically significant increase in the intervention group was noted in the scores of each item of self-management behavior compared with the scores at the baseline (*P*<.001). In the control group, the score of the item “self-isolate from families in home-based treatment” was lower than that at the baseline (*P*=.01), whereas scores of the item “maintain a balanced diet and ensure enough intake of high protein, vitamins, and minerals” (*P*=.002), item “stay in a good mood” (*P*=.002), item “cover your nose and mouth when coughing or sneezing” (*P*<.001), and item “wash hands properly” (*P*<.001) increased compared to the scores at the baseline. Source control is the most efficient measure to prevent infectious diseases. For item “self-isolate from families in home-based treatment,” the score was lower than that of any other behaviors in the intervention and control group. Moreover, it was found that the item “no intention to adopt such a behavior (namely, self-isolation)” had the most response, which may be attributed to the fact that not each patient with pulmonary TB is infectious and that the sputum culture result is needed to determine whether to carry out self-isolation or not. However, a comparison with the scores at the baseline shows that the scores of the intervention group increased (*P*<.001) and the scores of the control group decreased (*P=*.01). Extended duration of therapy was considered to be the main reason that patients did not want to self-isolate. However, efficient health education facilitates the choice to self-isolate.

Nutritional therapy is the basis of TB treatment. Each patient in our hospital was instructed to maintain a balanced diet, which increased the scores of the related behavior in both groups, and the scores of the intervention group were higher than those of the control group (*P=*.04). A positive mentality contributes to favorable treatment, and improvement of health brings pleasure to patients. In the 2 groups, the score of the item “stay in a good mood” increased with a higher increase in the intervention group, indicating a good effect of the intervention on emotion. The scores of the item “cover your nose and mouth when coughing or sneezing” and item “wash hands properly” showed a similar increase in both the groups. Pulmonary TB and COVID-19 have a similar route of transmission and prevention [32]. It is probable that the overall increase of the above two self-management behaviors can be attributed to the increased awareness of infectious disease prevention during the COVID-19 epidemic. This is consistent with the findings of Wang et al [33], that is, more people washed their hands with soap after touching contaminated objects and covered their mouths when coughing or sneezing as precautionary strategies during the initial stage of the COVID-19 epidemic in China. Further, the adherence of the patients in the control group gradually decreased over time, thereby presenting as a discontinuation of the drug and missed doses, which is consistent with the findings of Zomahoun et al [34], whereas the adherence of the patients in the intervention group increased significantly with daily medication reminders on the WeChat group. In addition, the intervention group had a higher revisit rate than the control group (*P*=.22) through review of medical records and consultation in WeChat, which indicated a positive effect of ITHBC-based mHealth intervention on patients’ adherence.

#### Secondary Outcomes

Compared with those at baseline, TB knowledge awareness, self-efficacy, social support, and degree of satisfaction with health education of the intervention group all increased significantly (*P*<.001), and the scores of the intervention group were higher than those of the control group (*P*<.001), thereby indicating that mHealth interventions for TB self-management based on ITHBC can improve behavior belief, self-efficacy, and social support of patients with TB. However, this finding is inconsistent with the results of most studies, which showed that single health education may result in increased patient resistance to change or engage in appropriate disease management strategies [35]. Knowledge is the foundation to change behavior. Researchers have confirmed the role of health education in promoting behavior beliefs, for example, a health education program significantly improved the health beliefs of participants with a history of opisthorchiasis [36]. Effective educational interventions are those that aim to modify patients’ behaviors rather than simply providing information. It is worth noting that before the intervention, the average score of the item “not spitting, covering your nose and mouth when coughing or sneezing, and wearing a mask can reduce the spread of TB” was 4.3, which was higher than the average score of 3.5-3.7 in other items. This showed that patients had a good understanding of how to reduce the spread of TB with or without health education.

Self-efficacy reflects the confidence of patients for disease control, and good self-efficacy helps change health behaviors. Health education might have helped patients to perceive risks and expect outcomes, thereby promoting the establishment of good beliefs. This study set different self-management goals for each patient according to different contexts through review at the baseline. This individualized education provided encouragement and helped to build confidence in defeating the disease. Moreover, volitional self-efficacy may be related to social support and follow-up. Some studies involved in other chronic diseases proved that partner support and home visits were useful ways to support patients [37,38]. ITHBC-based mHealth intervention pays more attention to the effect of social support on self-management behaviors. According to previous researches, patients often failed to continue treatment for the lack of family and community support [39,40]. In our study, the WeChat groups were used as tools to build partnerships among patients and between patients and medical staff, thereby providing positive social support and ultimately improving the self-efficacy of patients with TB. The intervention content was developed and optimized by multidisciplinary medical experts, and we gave it a pretest based on the needs of patients. Satisfaction with health education was explored from content, structure, and form, and it was shown that the score of each item in the intervention group was significantly higher than the score of the control group after the 3-month intervention. This illustrated that ITHBC-based mHealth allows researchers to provide a scientific, efficient, convenient, and easy way to understand health education and give timely responses for health confusion.

In the 3-month intervention, 2 patients in the control group dropped out, and 112 patients fulfilled the criterion of the smallest sample size in the study. Schulz and Grimes [41] suggest that losses to follow-up less than 5% usually have little impact, whereas losses greater than 20% raise serious flags about study validity. White and Thompson [42] advocated the use of mean imputation and missing-indicator method as a solution for practical purposes. The data in this study were missing at random, and we finally used mean substitution to fill and calculate the outcome of the control group. For results that had no significant differences from the conclusion, it could be concluded that lost visits exerted little effect on the conclusion and the conclusion was reliable.

It is not uncommon that some participants do not receive the intervention allocated by the randomization process. This study could not show whether the participants had actually adopted the recommended behaviors. Researchers have confirmed the gold standard of reporting is “intention-to-treat” analysis and according to the intervention that they actually received (per-protocol analysis) can rarely lead to differing results [25]. Therefore, in this study, we collected the outcomes of all the participants randomly assigned to the intervention group, even if some of the participants may not have adopted the recommended behaviors.

### Strengths and Limitations

This study had 2 strengths. First, we applied the ITHBC theory to study the self-management of patients with TB for the first time. Second, mHealth interventions did not face any restrictions during the COVID-19 pandemic and met the needs of the patients to the greatest extent. Moreover, healthy people are exposed to less risks owing to the application of mHealth [43,44].

This study had the following limitations. First, the intervention was only conducted in 1 hospital, which may restrict the general applicability of our results, and future studies should be conducted with larger sample sizes to confirm our results. Second, scores of TB knowledge awareness and friend support in both groups had significant differences at the baseline (*P*=.01), which may be due to the small sample size. Third, the short duration of observation (3 months) cannot prove the long-term efficacy. We plan to conduct studies with a long-term intervention (6 or 12 months) to focus on assessing the patient’s clinical efficacy and quality of life. Fourth, the nonrandom sampling method could result in some selection bias.

### Conclusion

In conclusion, ITHBC-based mHealth intervention may be a new promising therapeutic strategy for the management of TB for improving patients’ subjective initiative and self-management behaviors, which are beneficial for promoting compliance and the quality of prevention and control for pulmonary TB. In addition, mHealth provides an effective solution for outpatients without nursing care.

## Appendix

Multimedia Appendix 1 CONSORT-eHEALTH checklist (V 1.6.1).

Multimedia Appendix 2 Informed consent.

Multimedia Appendix 3 Screenshots of the health education in WeChat groups.

Multimedia Appendix 4 Topics of health education in the Knowledge and Belief section.

Multimedia Appendix 5 Questionnaire content.

## Abbreviations

DOT: directly observed therapy
ITHBC: Integrated Theory of Health Behavior Change
mHealth: mobile health
TB: tuberculosis
