# Supplementary material for: Effects of an mHealth Intervention for Pulmonary Tuberculosis Self-management Based on the Integrated Theory of Health Behavior Change: Randomized Controlled Trial
Source: JMIR Public Health Surveill. 2022 Jul 14;8(7):e34277. doi: 10.2196/34277 (PMC9335179; doi:10.2196/34277)
Supplement: Multimedia Appendix 2 [file publichealth_v8i7e34277_app2.pdf]

Multimedia Appendix 2. Informed consent.

Project unit: Harbin Chest Hospital
Project name: Research on the effect of mHealth intervention for pulmonary tuberculosis self-management based on the Integrated Theory of Health Behavior Change

Dear Ms. / Mr:
Thank you very much for participating in this study. We have just briefed you on the general picture of this study and you have the right to carefully understand the purpose, content and the process before it begins. And in the course of the study, you can always quit for whatever reason, but it will never affect your normal treatment.
1 、Purpose of research：
The study aims to explore and conduct a mHealth intervention based on the Integrated Theory of Health Behavior Change (ITHBC) in patients with pulmonary tuberculosis to increase their ability of self-care management.
2 、Research report：
Our study will last for 3 months, and the entire study process is free.
At the beginning of the project, you need to join the WeChat group, in which the pharmacists will provide popular science knowledge to you. Knowledge education contains basic knowledge, diet management, hygiene routines, and medication management. The topics of health education will be sent to the WeChat group in the form of articles, pictures, or videos. We will also organize communication and Q & A in the WeChat group, and remind you online or by telephone to take medication and check on time.
We need your cooperation. At baseline and at the end of the study , it takes about 10 minutes each time to complete the relevant scale according to their own real situation. In the process of filling out the questionnaire, the clinical pharmacist will help to explain the questionnaire if you need it to ensure the questionnaire was completed efficiently, but it is not instructive. You can always consult us or opt out.
3 、The principle of confidentiality：
We will definitely strictly protect your personal information data. All your information is used for this study and will be kept, not used in commercial transactions and never leaked.

If you agree to participate in this study, please sign your name in the space below. Thank you very much for your trust and cooperation!

Signature ：
Date：
