# Supplementary material for: Effects of an mHealth Intervention for Pulmonary Tuberculosis Self-management Based on the Integrated Theory of Health Behavior Change: Randomized Controlled Trial
Source: JMIR Public Health Surveill. 2022 Jul 14;8(7):e34277. doi: 10.2196/34277 (PMC9335179; doi:10.2196/34277)
Supplement: Multimedia Appendix 3 [file publichealth_v8i7e34277_app3.pdf]

Multimedia appendix 3. The screenshots of the health education in WeChat groups.

Screenshot 1 shows the WeChat group content of the patients in the intervention group.The pharmacists provided popular science knowledge to the patients in the intervention group and reminded the medication on time, and the patients responded.

Screenshot 2 shows the pharmacist communicating with the patients in the WeChat group, answering the questions during their treatment process and giving them encouragement.

To protect everyone's privacy, we have blurred the names and profile pictures.

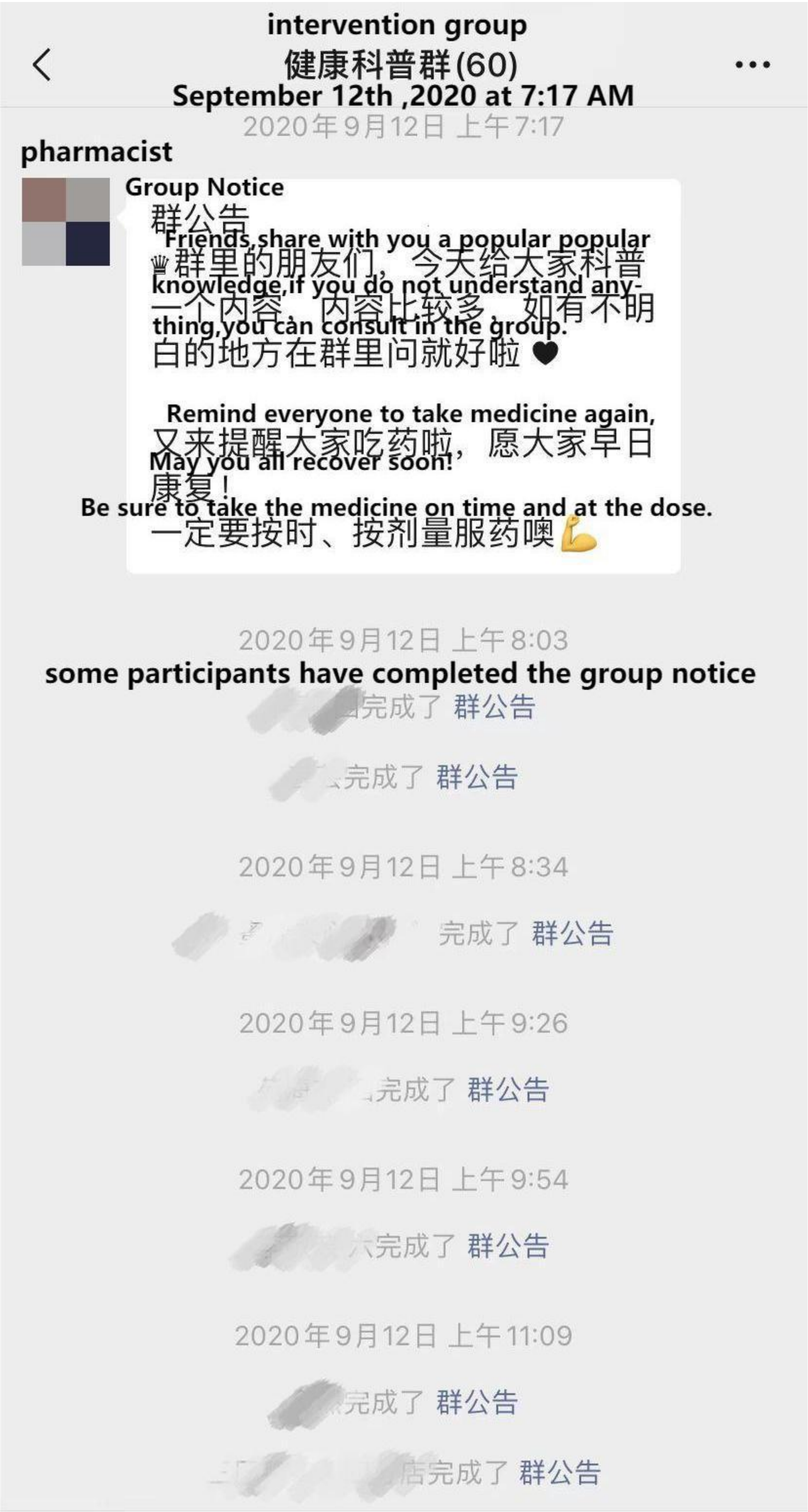
intervention group
健康科普群(60)
September 12th ,2020 at 7:17 AM
2020年9月12日 上午7:17
pharmacist
Group Notice
群公告
Friends,share with you a popular popular
♛群里的朋友们，今天给大家科普
knowledge,if you do not understand any-
一个内容，内容比较多，如有不明
thing,you can consult in the group.
白的地方在群里问就好啦 ❤
Remind everyone to take medicine again,
又来提醒大家吃药啦，愿大家早日
May you all recover soon!
康复！
Be sure to take the medicine on time and at the dose.
一定要按时、按剂量服药噢💪
2020年9月12日 上午8:03
some participants have completed the group notice
完成了 群公告
完成了 群公告
2020年9月12日 上午8:34
完成了 群公告
2020年9月12日 上午9:26
完成了 群公告
2020年9月12日 上午9:54
完成了 群公告
2020年9月12日 上午11:09
完成了 群公告
完成了 群公告

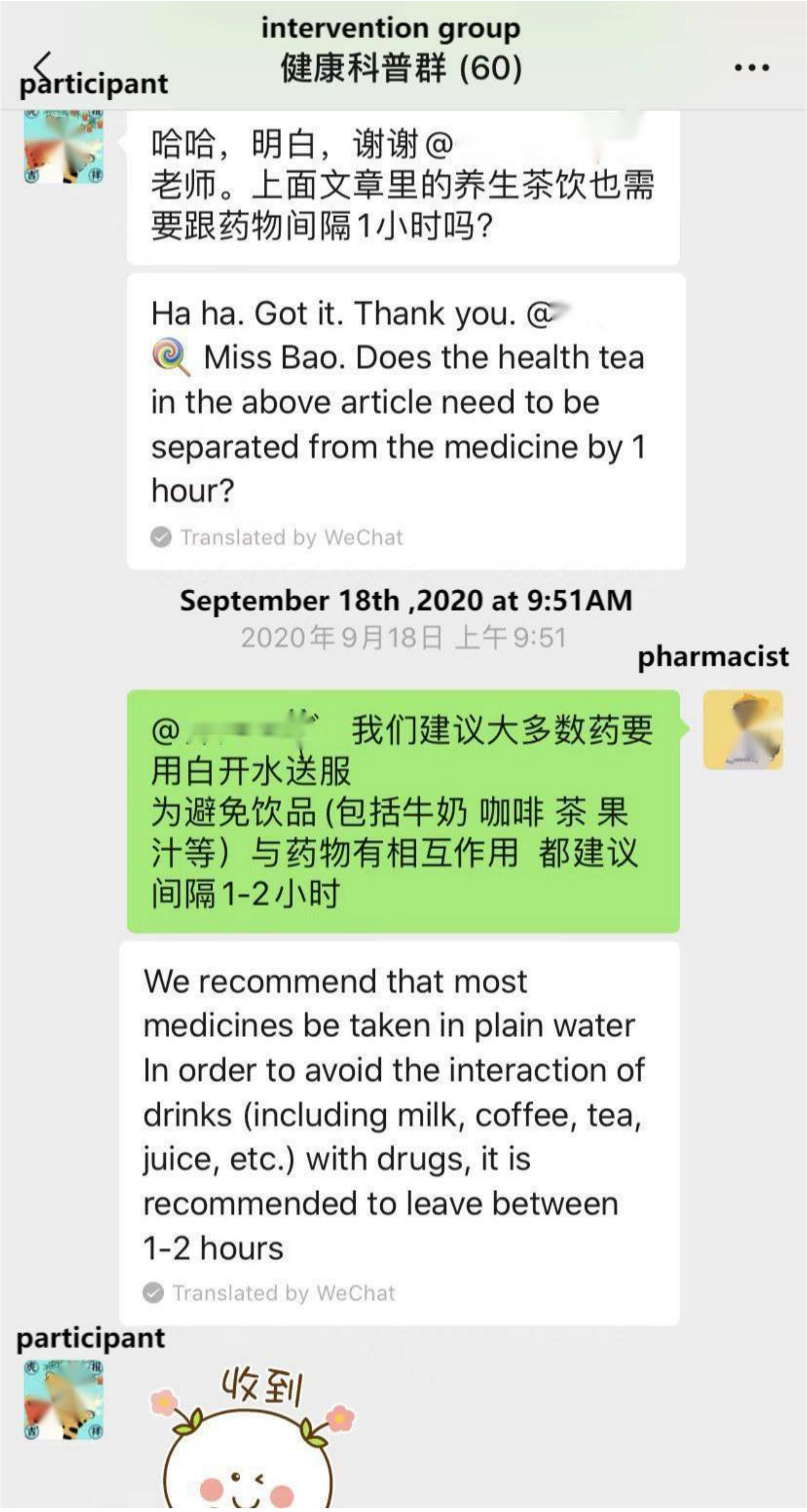
intervention group
健康科普群 (60)
participant
哈哈，明白，谢谢@
老师。上面文章里的养生茶饮也需要跟药物间隔1小时吗?
Ha ha. Got it. Thank you. @
Miss Bao. Does the health tea in the above article need to be separated from the medicine by 1 hour?
Translated by WeChat
September 18th ,2020 at 9:51AM
2020年9月18日 上午9:51
pharmacist
@ 我们建议大多数药要用白开水送服
为避免饮品(包括牛奶 咖啡 茶 果汁等）与药物有相互作用 都建议间隔1-2小时
We recommend that most medicines be taken in plain water In order to avoid the interaction of drinks (including milk, coffee, tea, juice, etc.) with drugs, it is recommended to leave between 1-2 hours
Translated by WeChat
participant
收到
