# Supplementary material for: Effects of an mHealth Intervention for Pulmonary Tuberculosis Self-management Based on the Integrated Theory of Health Behavior Change: Randomized Controlled Trial
Source: JMIR Public Health Surveill. 2022 Jul 14;8(7):e34277. doi: 10.2196/34277 (PMC9335179; doi:10.2196/34277)
Supplement: Multimedia Appendix 4 [file publichealth_v8i7e34277_app4.pdf]

Multimedia Appendix 4. Topics of health education in the Knowledge and Belief section.

Knowledge	Belief
**Base knowledge**	What are the effects on my body, life, family if my disease worsens?
What is pulmonary tuberculosis?	Tell patients the development of their disease to relieve doubts and fears.
What are common symptoms of pulmonary tuberculosis?	Tell patients the effectiveness of treatment and build their confidence by offering and analyzing cured cases.
How pulmonary tuberculosis spreads?	If I keep a positive outlook during therapy, I may get an excellent prognosis.
What the difference between pulmonary tuberculosis and COVID-19?	What difficulties I may be faced with when adopting measures suggested? And how to overcome?
Can I be cured of pulmonary tuberculosis?	
**Diet management**	
How should I manage my diet during treatment (e.g., nutrition, avoid alcohol, or potential interaction with drugs)	
**Hygiene routines**	
How can I prevent the spread of pulmonary tuberculosis? (including isolation, respiratory etiquette, handwashing, room ventilation, and disinfection strategies)	
**Medication management**	
What are the trade names of drugs for medication?	
How to use drugs properly? (e.g., dose, frequency, or length of therapy)	
What adverse effects may occur and how to deal with them?	
How to restore medications?	
When should I revisit for checkups?	
What conditions may occur if I don't take medicine as directed?	
