# Supplementary material for: Effects of an mHealth Intervention for Pulmonary Tuberculosis Self-management Based on the Integrated Theory of Health Behavior Change: Randomized Controlled Trial
Source: JMIR Public Health Surveill. 2022 Jul 14;8(7):e34277. doi: 10.2196/34277 (PMC9335179; doi:10.2196/34277)
Supplement: Multimedia Appendix 5 [file publichealth_v8i7e34277_app5.pdf]

Multimedia Appendix 5. Questionnaire content.

Questionnaire 1: Demographic Information.:

1. Your name: [fill empty title]*

____

2. Your gender: [single choice questions]*

○ male

○ female

3. Your age: [single choice questions]*

○ under 18 years of age

○18~25

○26~30

○31~40

○41~50

○51~60

○ above 60

4. race group [single choice questions]*

○ Han

○ Hui

○ zhuang

○ Tibetan

○ Miao

○ Man

○ other

5. Religious beliefs [single choice questions]*

○ yes

○ no

6. Marital status [single choice questions]*

○ unmarried

○ married

○ divorce

○ widowed

7. Family per capital monthly income [multiple-choice questions]*

○ no income

○ 1000 yuan and below

○ 1000- 1999 yuan

○ 2000-2999 yuan

○ 3000-3999 yuan

○ 4000-4999 yuan

○ 5000 and above

8. Usual place of residence [multiple choice]*

○ rural

○ urban

9. An industry you are currently engaged in or prior to retirement [multiple choice]*

○ organ / business unit personnel

○ skilled person
○ enterprise unit personnel
○ business, service sector personnel
○ agroforestry, Lin, animal husbandry, Fisher, water industry producer
○ production, transport equipment operators and personnel involved
○ military
○ student
○ other practitioners and lay people
10. Educational level [single choice]*
○ no formal education
○ primary school
○ junior middle school
○ technical secondary school
○ high school
○ University specialized
○ college undergraduate
○ master graduate student
○ doctor graduate student
11. Residence status for nearly two months [multiple-choice questions]*
□ spouse
□ children
□ parent
□ grandchildren
□ grandparents / maternal grandparents
□ living alone
□ nursing home / hospital
□ school / unit
□ others
12. Have any of your relatives or friends been (ever or current) infected with tuberculosis? [single choice]*
○ yes
○ no

Questionnaire 2: Health scale

Questions below are to ask how you feel about your health, how you feel and how well you are able to carry out your daily life. If you do not grasp how to answer the question, try to select the best answer.

1. your name is : 【fill in the blank】

____

2. The following questions can help us understand your social support, please choose the answer that best suits your thinking [matrix single choice questions]*

	Strongly disagree	very disagree	Slightly disagree	neutral	Slightly agree	very agree	Strongly disagree
Some people (relatives, neighbors, and colleagues) will be on my side when I have problems	○	○	○	○	○	○	○
I am able to share joy and blues with some people (relatives, neighbors, and colleagues)	○	○	○	○	○	○	○
My family can help me practically and specifically	○	○	○	○	○	○	○
I was able to emotionally aid and support from the family when needed	○	○	○	○	○	○	○
When I have difficulty some people	○	○	○	○	○	○	○

(relatives, neighbors, and colleagues) are the real source of comfort to me							
My friends can really help me	◦	◦	◦	◦	◦	◦	◦
I can rely on my friends when difficulties occur	◦	◦	◦	◦	◦	◦	◦
I can talk to my family about my difficulties	◦	◦	◦	◦	◦	◦	◦
My friends can share joy and blues with me	◦	◦	◦	◦	◦	◦	◦
There are certain people (relatives, neighbors, and colleagues) in my life who care about my feelings	◦	◦	◦	◦	◦	◦	◦
My family was willing to assist me with all decisions	◦	◦	◦	◦	◦	◦	◦
I can talk to friends about my difficulties	◦	◦	◦	◦	◦	◦	◦

3. The following questions can help us understand your knowledge of lentigines, select the answer that most closely resembles your inner idea [matrix single choice questions]*

	totally	disagree	uncertain	agree	totally

	disagree				agree
Tuberculosis is a chronic infectious disease that seriously endangers people's health for a long time	◦	◦	◦	◦	◦
Tuberculosis is primarily transmitted via the respiratory tract, and everyone may be infected	◦	◦	◦	◦	◦
Coughing and expectoration for more than 2 weeks, tuberculosis should be suspected, and prompt medical visits should be made	◦	◦	◦	◦	◦
The spread of tuberculosis can be reduced by indiscriminate spitting of sputum, and masking of the nose during coughing and sneezing	◦	◦	◦	◦	◦
Standardizing treatment to the full course of care, the vast majority of patients can be cured and may also avoid contagion to others	◦	◦	◦	◦	◦

4． Here are the stages of behavior you are now in, and select the one that best suits your situation: [matrix single question]*

	It is not intended to act on	Be aware of the importance of the action and intend to act	Be aware of the importance of the behavior and decide to take the behavior near or immediately	The action had been taken and persisted uninterrupted	Action taken but failed to adhere or interrupted

Quarantine yourself from families in home- based treatment	◦	◦	◦	◦	◦
keep good ventilation in your room and adopt disinfection measures in daily life	◦	◦	◦	◦	◦
maintain a balanced diet and ensure the intake of protein, vitamins and minerals	◦	◦	◦	◦	◦
stick to daily exercise	◦	◦	◦	◦	◦
go to bed and wake up early, choose alternate work with rest	◦	◦	◦	◦	◦
stay in a good mood	◦	◦	◦	◦	◦
cover your nose and mouth when coughing or sneezing	◦	◦	◦	◦	◦
properly choose and wear a mask	◦	◦	◦	◦	◦
wash hands properly	◦	◦	◦	◦	◦
adopt infection	◦	◦	◦	◦	◦

control measures outside					
focus your own symptoms and adverse drug reactions	◦	◦	◦	◦	◦
do not change regimen easily, withdrawal and change dressing	◦	◦	◦	◦	◦
regularly follow up and timely communicate with doctors	◦	◦	◦	◦	◦

5. The following headings give an idea of how satisfied you are with online popular science once you have entered wechat group, please select the one that best fits your situation [matrix bullet questions]*

	Very dissatisfied	disagree	More disagree	commonly	More agree	agree	Very satisfied
Online popular science health information is reliable	◦	◦	◦	◦	◦	◦	◦
Online popular science health information is comprehension	◦	◦	◦	◦	◦	◦	◦
Popular online science health messages are clear and easy to understand	◦	◦	◦	◦	◦	◦	◦
Online popular science health messages are well structured	◦	◦	◦	◦	◦	◦	◦

Access to health information online is straightforward	◦	◦	◦	◦	◦	◦	◦
I can quickly adapt to online popular science	◦	◦	◦	◦	◦	◦	◦
I can easily find the information needed in popular science online	◦	◦	◦	◦	◦	◦	◦
Online popular science is practical	◦	◦	◦	◦	◦	◦	◦
Online popular science meets my health information needs	◦	◦	◦	◦	◦	◦	◦
Online science popularization can always answer my questions	◦	◦	◦	◦	◦	◦	◦

The following question is to understand your recent confidence in dealing with some of your questions, and you choose it according to your actual situation (numerical meaning: " 1 "means no confidence at all, and so on, the larger the number, the more confident, "10 " means absolutely confident)

6. How confident are you in managing this fatigue since the fatigue caused by your disease prevents you from doing what you want to do on your own? [Single choice]*

○Not at all confident

○two ○three ○four ○five ○six ◦ seven ○eight ○nine

○ completely confident

7. How confident are you in controlling this physical discomfort or pain due to your disease that prevents you from doing what you want to do?【Single choice】

○Not at all confident

○two ○three ○four ○five ○six ◦ seven ○eight ○nine

○ completely confident

8.How much confidence do you feel in controlling this emotional depression because the emotional depression caused by your illness prevents you from doing what you want to do?【Single choice】

○No confidence at all

○2 ○3 ○4 ○5 ○6 ○7 ○8 ○9

○ Absolutely confident

9.Any symptoms or health problems that prevent you from doing what you would like to do on your own, and how confident are you in controlling these symptoms or health problems?【Single choice】

○No confidence at all

○2 ○3 ○4 ○5 ○6 ○7 ○8 ○9

○ Absolutely confident

10.How confident are you in taking these self-care behaviors in order to reduce the number of visits to a doctor that can lead to certain self-care behaviors? [Single choice]*

○No confidence at all

○2 ○3 ○4 ○5 ○6 ○7 ○8 ○9

○ Absolutely confident

11.How confident are you in doing something other than using medication in order to mitigate the impact the disease has on your daily life?【Single choice】

○No confidence at all

○2 ○3 ○4 ○5 ○6 ○7 ○8 ○9

○ Absolutely confident
